# Impact of the COVID-19 pandemic on suicidal attempts and death rates: a systematic review

**DOI:** 10.1186/s12888-022-04158-w

**Published:** 2022-07-28

**Authors:** Malshani L. Pathirathna, Hapugahapitiye Mohottalage Renu Kalhari Geethani Nandasena, Atapattu Mudiyanselage Muditha Piumali Atapattu, Ishanka Weerasekara

**Affiliations:** 1 Department of Nursing, Faculty of Allied Health Sciences, University of Peradeniya, Peradeniya, 20400 Sri Lanka; 2 Department of Physiotherapy, Faculty of Allied Health Sciences, University of Peradeniya, Peradeniya, 20400 Sri Lanka; 3 College of Health Medicine and Wellbeing, The University of Newcastle, Newcastle, Callaghan 2308 Australia

**Keywords:** COVID-19 pandemic, Lockdown, Mental health, Suicide, Suicidal ideation, Suicide attempted, SARS-CoV-2 infection

## Abstract

**Background:**

The COVID-19 pandemic has exacerbated the risk factors associated with suicidal behavior and thus, prioritizing its prevention is recommended.

**Methods:**

This study systematically reviewed the global evidence on the incidence of suicide/suicidal attempts and the trend in suicidal rates during the COVID-19 pandemic. Cross-sectional and cohort studies investigating the outcomes of suicidal death and suicidal attempts at any setting during the COVID-19 pandemic were searched in Medline, Embase, and PsycINFO databases for papers published from December 2019 to May 2021.

**Results:**

Out of 1052 studies18 studies with 12,746 suicidal attempts and 33,345 suicidal deaths were included in the final analysis. The mental health impact of social distancing, COVID-19 quarantine, and financial crises due to loss of employment were associated risk factors with suicide and/or suicidal attempts during the COVID-19 pandemic. Six common thematic recommendations for preventing suicidal deaths and suicidal attempts were identified.

**Conclusions:**

Unexpected behavior changes during the COVID-19 pandemic may have contributed to the increasing trend of suicidal attempts reported. Domestic conflicts and violence, financial loss, anxiety and depression, and pre-existing mental health condition/s should be considered in preventing suicidal attempts and deaths secondary to the COVID 19 pandemic. Early detection and timely intervention for individuals with suicidal behavior is crucial and collated recommendations in the current study can be utilized for those preventive interventions. More systematic suicide risk screening process should be introduced who are at risk, along with an evidence base prevention approach.

## Introduction

COVID-19 is an infectious disease caused by SARS-CoV-2 [1]. The first case of COVID-19 was reported in Wuhan, China, on December 31st, 2019, and afterward continued to spread across nearly 200 countries, causing an infection fatality rate of ~ 0.15% [2]. Considering the alarming levels of spread and severity, World Health Organization (WHO) declared it a pandemic on March 11th, 2020 [3]. Almost all the countries in the world provoked public health measures such as home confinement, closure of schools and universities, travel restrictions, and limit/ withhold social and physical distancing, while some countries declared it a public health emergency [4]. Many countries had to impose periods of lockdowns as an attempt to limit the spread of SARS-CoV-2. By early April 2020, more than one-third of the global population was under some form of movement restriction [5]. These strategies implemented in different countries intensively have caused substantial social and economic disruptions to individuals and the whole community [6]. While this disease has already directly impacted the physical health of millions of people, it is also causing mental health problems globally [7–9]. The evidence on the mental health harms caused by the response to COVID-19 found to be overwhelming, and studies on the general public revealed lower psychological well-being and higher scores of anxiety and depression compared to before COVID-19 [10]. The WHO also expressed its concern on the effect of COVID-19 on mental health and psycho-social consequences of an individual and it is estimated that COVID-19 pandemic triggers 25% increase in prevalence of anxiety and depression worldwide [11]. As new measures imposed in many countries such as self-isolation and quarantine, which affects day-to-day activities, routines, and livelihoods of people, they may lead to an increase in loneliness, anxiety, depression, insomnia, harmful alcohol and drug use, and self-harm or suicidal behaviour [12]. There is substantial evidence to demonstrate the deterioration of mental health among people during and after the COVID-19 pandemic compared to the pre-COVID-19 period [13–16]. Stack and Rockett (2021) discovered an increased suicide rate during the Spanish flu epidemic in 43 large cities, which was connected to the degree of social distancing, independent of the influenza fatality rate [17]. According to WHO, Every year more than 700,000 people die due to suicide [18]. However, there was no consistent evidence of a rise in suicide during and post-COVID era [19]. In this context, the research evidence on the effect of COVID-19 on suicides and suicidal attempts began to expand very rapidly. It is, therefore, timely and important to collate the global evidence on the incidence of suicide/suicidal attempts and the trend in suicidal rates during the COVID-19 pandemic. Thus, this study aimed to systematically review the available literature on (i) the incidence of suicidal attempts, suicidal deaths, and the trends in suicidal rates during the COVID-19 pandemic (ii) the risk factors for suicidal attempts and suicidal deaths during the COVID-19 pandemic, and (iii) the recommendations in preventing suicidal attempts or suicidal deaths during the COVID-19 pandemic.

## Methods

### Information sources and search strategy

The protocol for this systematic review was registered with the International Prospective Register of Systematic Reviews on May 5th 2021 (CRD42021253347). The Medline, Embase, and PsycINFO databases were searched for relevant studies from December 2019 to May 2021. The search strategy included a combination of keywords for COVID-19 and suicide/suicidal attempts. The language was restricted to English.

### Study selection

Eligibility criteria for study inclusion were as follows: (1) studies that investigated suicidal deaths/ attempts of humans during COVID-19 pandemic (2) published after December 2019 and (3) adequately described the data on outcomes of suicidal death and suicidal attempts at any setting. Studies were excluded if any of the following criteria were noticed: (1) studies in languages other than English (2) study designs such as case studies, case reports, commentaries, editorials, letters to editor, reports, reviews, and systematic reviews, and (3) non-peer-reviewed articles and conference abstracts. The updated Preferred Reporting Items for Systematic Reviews and Meta-Analyses (PRISMA) guidelines were followed. Also the review has conducted in accordance with Cochrane handbook for systematic reviews [20]. The studies yielded during the search were exported to the Endnote (EndNote X9. 3. 3 version) reference manager software. Following the removal of duplicates, screening against the inclusion and exclusion criteria was undertaken by two independent individuals in two stages; 1) title and abstract screening, and 2) full-text screening in Covidence systematic review software (Veritas Health Innovation, Melbourne, Australia). Any discrepancies were resolved by consensus or by consultation with a third reviewer.

### Data extraction

Two individuals extracted the data independently from the included full texts into an Excel spread sheet and cross-checked for further accuracy. Any discrepancies were resolved by consensus. Extracted information included the publication details (year, authors, country), characteristics of the studied sample (age, sex, and studied population), outcome variables (suicide deaths and suicidal attempts, method of suicide, previous history of suicide, and other risk factors reported), and recommendations to prevent future suicidal deaths or attempts.

### Assessment of the quality of the studies

We assessed the quality of the included articles using the Quality Assessment Tool for Observational Cohort and Cross-Sectional Studies [21]. The checklist contained fourteen items, including 1) Was this paper’s research question or objective clearly stated? 2) Was the study population clearly specified and defined? 3) Was the participation rate of eligible persons at least 50%? 4) Were all the subjects selected or recruited from the same or similar populations (including the same time period)? Were inclusion and exclusion criteria for being in the study prespecified and applied uniformly to all participants? 5) Was a sample size justification, power description, or variance and effect estimates provided? 6) For the analyses in this paper, were the exposure(s) of interest measured prior to the outcome(s) being measured? 7) Was the timeframe sufficient so that one could reasonably expect to see an association between exposure and outcome if it existed? 8) For exposures that can vary in amount or level, did the study examine different levels of the exposure as related to the outcome (e.g., categories of exposure or exposure measured as continuous variable)? 9) Were the exposure measures (independent variables) clearly defined, valid, reliable, and implemented consistently across all study participants? 10) Was the exposure(s) assessed more than once over time? 11) Were the outcome measures (dependent variables) clearly defined, valid, reliable, and implemented consistently across all study participants? 12) Were the outcome assessors blinded to the exposure status of participants? 13) Was loss to follow-up after baseline 20% or less? 14) Were key potential confounding variables measured and adjusted statistically for their impact on the relationship between exposure(s) and outcome(s)? Two reviewers assessed the articles against each of the 14 items independently and then cross-checked them. Any discrepancies were resolved by consensus.

### Data analysis

Descriptive statistics were used to report the findings of included studies according to the research objectives. Tables and graphs were used accordingly to present the details of the publication, characteristics of the sample and outcome variables. Common themes were identified and reported in presenting future recommendations for preventing COVID-19 related suicidal deaths and attempts.

## Results

**Fig. 1:**
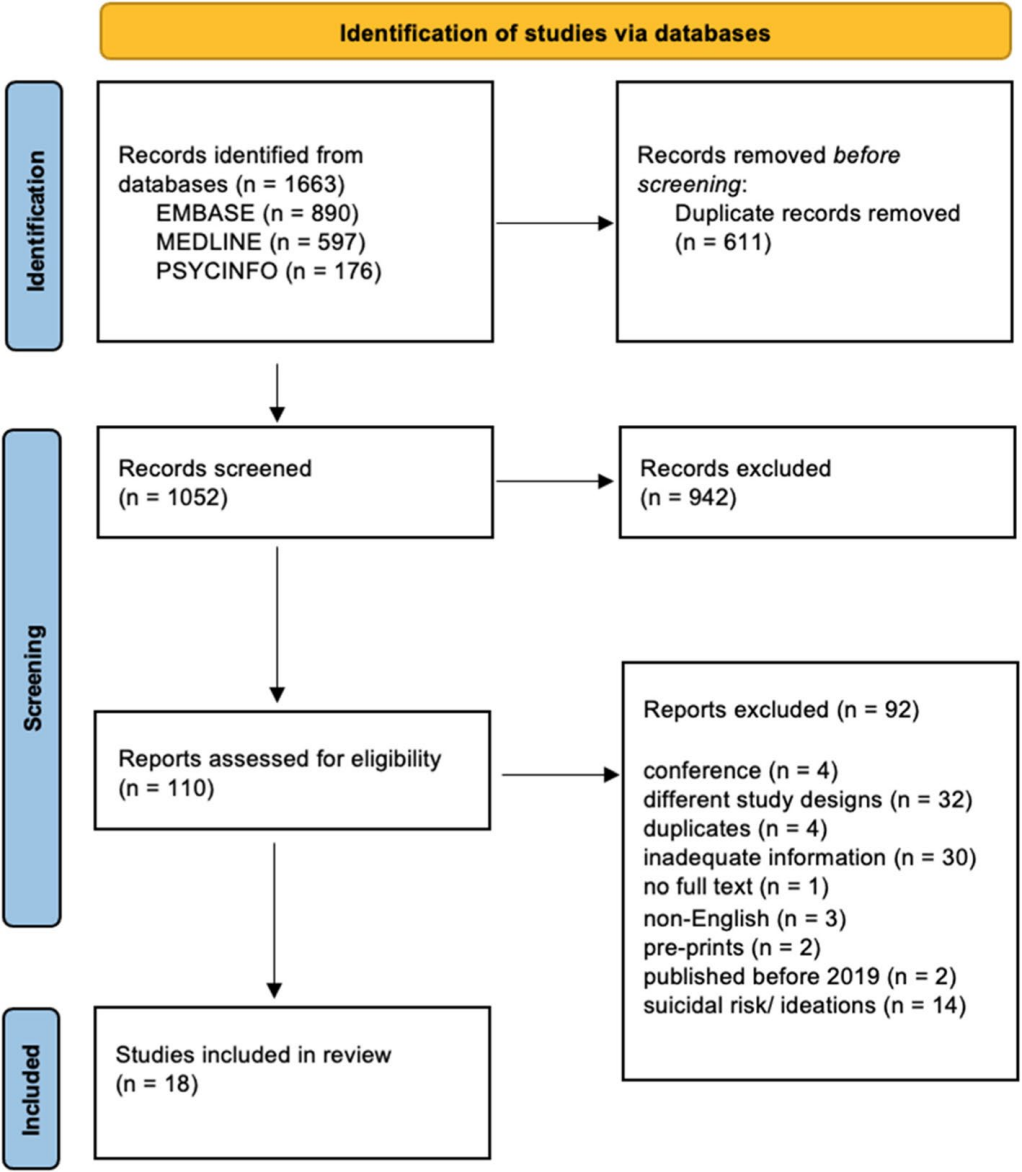
PRISMA flow chart of included studies

Based on the database search, 1663 articles published between December 2019 and May 2021 were retrieved, and 611 duplicates were removed. Out of 1052 studies, 942 were excluded during the title and abstract screening. Subsequently, 110 full articles were screened at the full-text screening stage, and 18 were included. The main reasons for exclusions were different study designs, suicidal risk/ ideations, and inadequate information. The study flow chart outlines the detailed review process (Fig. 1).

## Characteristics of included studies

The main characteristics of the included studies are listed in Table 1. Some of the studies’ studied samples were composed only of suicidal attempts or suicidal deaths [22–29], while some of the studies included a wider sample to see the incidence of suicidal deaths and suicidal attempts [30–38]. The sample sizes of the studies that included only the suicidal deaths or suicidal attempts were ranged from 53 to 17,794. Sixteen (16) studies (88.9%) had a cross-sectional design and two studies [24, 26] have followed cohort design. Out of 18 studies included, 8 (44.4%) were from the United State of America [22, 24, 26, 30, 32, 34, 36, 38], 4 (22.2%) from Japan [25, 27, 29, 35], 2 (11.1%) from India [23, 37] and 1 (5.6%) each from Austria [31], Canada [33], German [28] and Nepal [39].

**Table 1:** Characteristics of included studies

Study (author and year)	Geography	Data collection period	Source of information	Design of the study	Studied population/occupation	Characteristics of the study sample			
						**Sample size†**	**Age**	**Female/ male n (%)**	**Occupation**
Ammerman et al., 2021 [30]	USA	April 03 – 04, 2020	Online survey	Cross-sectional study	Amazon Mechanical Turk (mTurk) population	907	Mean (SD) 36.43 (11.02)	Male 511 (56.3); Female 387 (42.6); Trans-gender 4 (0.4); Preferred not to answer 5 (0.6)	Amazon Mechanical Turk (mTurk)
Bergmans and Larson 2021 [22]	USA	March 10th—October 31st, 2020	Electronic medical records	Retrospective cross-sectional study	Daily ED encounters for suicide attempts and intentional self-harm to the University of Michigan Health System	1348	Mean (SD) 27.9 (14.3)	Male 533 (39.5); Female 815 (60.5);	NR
Carlin et al., 2021 [31]	Austria	March 16th—May 15th, 2020	Admission data at Trauma Resuscitation room of the Level 1 Trauma Center of the Medical University of Vienna-Austria	Retrospective cross-sectional study	Admissions to the level 1 Trauma Centre of the Medical University of Vienna	65	Mean (SD) 38.7 (12.2)	Male 17 (73.9); Female 06 (26.1)	NR
Chiba et al., 2020 [32]	State of California, USA	March 20th—June 30th, 2020	Trauma registry of Los Angeles County and University of Southern California Medical Center	Retrospective cross-sectional study	Trauma admissions to the Los Angeles County and University of Southern California Medical Center	1,202	Median (IQR) 40 (27–57)	Male 927 (77.2) Female 275 (22.8)	NR
Choudhury, 2020 [23]	India	March 24th—May 31st, 2020	Police records- Lucknow, India	Retrospective cross-sectional study	Suicidal deaths in Lucknow, India	59	NR	Male 33(55.9); Female 26(44.4)	Business /self-employed 10(16.9%); Daily wages worker 15(25.4%); Govt. employee 3(5.0%); Domestic help 9(15.2%); Housewife 10(16.9%); Farmer 4(6.7%); Student 5(8.4%); Health care worker 3(5.0%)
Daly et al*.,* 2021[33]	Canada	May 14 – 29, 2020	Online survey	Cross-sectional study	Canadian adults	3000	Range 18 to 55 +	Male 1460 (48.7); Female 1519 (50.6); Other 21 (0.7)	NR
Faust et al*.,* 2021 [24]	USA	January—July 2020	Health Registry of Vital Records and Statistics	Cohort study	Suicide death data from the Massachusetts Department of Health Registry of Vital Records and Statistics	139	Mean 48.6	Male 105 (75.5); Female 34 (24.5)	NR
Hill et al*.,* 2021 [34]	Texas, USA	January to July 2020	Electronic health records data for suicide-risk screens of an emergency department in a major metropolitan area in Texas	Cross-sectional study	Youth aged 11 and older who presented to the ED within any of three connected pediatric hospitals for any presenting complaint	12,827	NR	Male 5257 (41%) Female 7570 (59%)	NR
Holland et al*.,* 2021 [35]	Japan	December 29th, 2019—October 10th, 2020	Centres for Disease Control and Prevention’s National Syndromic Surveillance Program	Cross-sectional study	Patients who visited USA Emergency Departments for mental health conditions, suicide attempts, all drug overdose, opioid overdose, intimate partner violence, and child abuse and neglect	1,872,945	NR	Female 1,013,585 (0.54); Male 859,360 (0.46)	NR
Isumi et al., 2021 [25]	Japan	March–May 2020	Public data on suicide statistics compiled by the Ministry of Health, Labor and Welfare	Cross-sectional study	Suicidal death among children and adolescents under 20 years of age	NR	NR	NR	NR
Mitchell & Li 2021 [26]	USA	March 10th—May 20th, 2020	Connecticut Office of the Chief Medical Examiner	Retrospective cohort study	Residents in Connecticut State	74	Mean (SD) 50.8 (18.5)	Male 55 (74.3); Female 19 (25.7)	NR
Nomura et al., 2021 [27]	Japan	January—September 2020	Monthly mortality data from the National Police Agency	Cross-sectional study	Suicidal deaths in Japan	15,066	NR	Male 10,239 (68); Female 4827 (32)	NR
Ontiveros et al*.*, 2021 [36]	USA	March–May, 2020	California Poison Control System database	Cross-sectional study	Calls received to the California Poison Control System regarding suicide attempts (suicidal ingestion)	5807	Range 12 to 70 +	Male 2149 (37); Female 3658 (63)	NR
Radeloff et al*.*, 2021 [28]	German	January 1st—September 30th, 2020	City of Leipzig’s cause of death statistics	Cross-sectional study	Data on suicide events in a major city in Germany- Saxony	53	NR	NR	NR
Sakamoto et al*.,* 2021 [29]	Japan	January—December 2020	National data on suicide deaths compiled by the National Police Agency	Cross-sectional study	National data of the number of individuals who died of suicide – Japan Self-employed-1019 (6%); Emplyed-5231 (31%); Student-1576 (9.4%); Unemployed-8095 (48%); Home maker-938 (5.6%) Occupational Data missing ( 935)	17,794	Range < 30 to ≥ 70	Male 11,779 (66.2); Female 6015 (33.8)	NR
Sengupta et al., 2020 [37]	India	January 25- April 24, 2020	Inquest reports, bed head tickets, injury reports and other relevant documents. Information was gathered from the deceased’s close relatives, friends, police and other available persons	Cross-sectional study	Autopsies conducted in the Department of Forensic Medicine and Toxicology, Cooch Behar Government Medical College and Hospital	334	Range 11 to 70 +	Male- 60 (54.5); Female-50 (45.5)	Farmers- 12 (10.9%) Labourer-16 (14.6%) Service men-5 (4.5%) Businessmen-12 (10.9%) Stduents-10 (9.1%) Housewives- 34 (30.9%) Unemployed-16 (14.6%) Unknown-5 (4.5%)
Shrestha et al., 2021 [39]	Nepal	March 24—June 23, 2020	The electronic medical records of a Hospital	Cross sectional study	Patients presented to the ED of Dhulikhel hospital-Kathmandu University Hospital (DH-KUH) -Nepal	55	Median (95% CI) 29.7 (26.2–33.3)	Male 21 (38.2); Female 34 (61.8)	NR
Yeates et al*.,* 2021 [38]	USA	January 1st—June 30th, 2020	Trauma Center Records	Cross-sectional study	Southern California trauma population	12,741	NR	NR	NR

*†Sample size presented relevant to the data collection period mentioned in the same table, SD Standard deviation,  NR* Not reported

## Incidence and the trends of suicide/suicidal attempts during the COVID-19 pandemic

12,746 suicidal attempts and 33,345 suicidal deaths were reported during the COVID-19 pandemic in the included studies. One other study has not reported separate data for suicidal attempts and deaths (*n* = 55), while one has presented rates. Because of the different durations used for data collection during the COVID-19 pandemic and the previous year (for non-COVID time comparison), it was unable to estimate or compare the suicidal incidence rate or to pool the data together for a meta-analysis. 16.7% (*n* = 3) of studies have no reported data on suicidal attempts or suicidal deaths trends. Regarding trends in suicidal attempts during the COVID-19 pandemic, an increasing trend was reported in 22.2% (*n* = 4) studies, while a decreased trend was reported in 11.1% (*n* = 2) studies. 5.6% (*n* = 1) of studies reported no increased or decreased trend in suicidal attempts. An increasing trend of suicidal deaths during the COVID-19 pandemic was found in 16.7% (*n* = 3), while decreased in 5.6% (*n* = 1) studies. 16.7% (*n* = 3) of studies reported no increased or decreased trends in suicidal deaths. Finally, 5.6% (*n* = 1) of studies reported decreased trends during the crisis but increased after the immediate crisis had passed (Table 2).

**Table 2:** The impact of the COVID-19 pandemic on suicidal attempts, death rates and trends

Study (author and year)	Suicidal deaths (SD)/ suicidal attempts (SA) during COVID-19	Number of events during the corresponding period of the previous year (2019)	Method of suicide/ suicidal attempts	Previous history of attempts	The trend of suicides/ suicidal attempts during COVID pandemic
Ammerman et al., 2021 [30]	*SA*: 44 (4.9%)	NR	NR	NR	NR
Bergmans and Larson 2021 [22]	*SA and Intentional self-harm:* 1348	October 1st, 2015 to March 9th, 2020: 9405	NR	NR	*SA and Intentional self-harm:* Decreased
Carlin et al., 2021 [31]	*SA:* 23 (35.4%)	2019: 8	Jump from a height 10 (43.5%); Jump in front of a moving object 2 (8.7%); Cutting 6 (26.1%); Driving off the street 1 (4.3%); Self-immolation 1 (4.3%); Hanging 2 (8.7%); Ingestion of poison, harmful substance etc. 1 (4.3%)	NR	*SA:* Increased
Chiba et al., 2020 [32]	*SA:* 36 (3%)	2019: 26	NR	NR	*SA:* Increased
Choudhury, 2020 [23]	*SD*: 59	NR	Hanging 55(93.2%); Poisoning 2 (3.3%); Drowning 2 (3.3%)	NR	NR
Daly et al*.,* 2021 [33]	*Deliberate self-harm:* 48 (1.6%)	NR	NR	NR	NR
Faust et al*.,* 2021 [24]	*SD:* 139	2019: 166	NR	NR	*SD:* No change
Hill et al*.,* 2021 [34]	*SA: 286 (2.2%)*	2019: 268	NR	NR	*SA*: Increased during some months (corresponds to COVID-19–related stressors and community responses were heightened)
Holland et al*.,* 2021 [35]	*SA*: 5029	2019; 4614	NR	NR	*SA:* Increased
Isumi et al., 2021 [25]	*SD:* Only the rates presented- March 0.229, April 0.201, May 0.216 per 100,000)	NR	NR	NR	*SD:* No change
Mitchell & Li 2021 [26]	*SD*: 74 (Age adjusted suicidal rate in 2020- 9.4 per 100,000 persons)	2014 to 2019; 495 (Total)	Suffocation- 35 (47.3%) Firearm- 21 (28.4%) Poisoning- 13 (17.6%) Other—5 (6.8%)	NR	*SD:* Decreased
Nomura et al., 2021 [27]	*SD:* 15,066	2019; 15,520	NR	NR	*SD:* Decreased during the time of crisis but increased after the immediate crisis has passed
Ontiveros et al*.*, 2021 [36]	*SA*: 5807	2018 and 2019; 13,800 (Total)	Ingestion of poison	NR	*SA:* Decreased
Radeloff et al*.*, 2021 [28]	*SD:* 53	2010 to 2019; 590 (Total)	NR	NR	*SD:* Not changed
Sakamoto et al*.,* 2021 [29]	*SD:* 17,794	2016 to 2019; 12,398 (total of monthly mean number from)	NR	NR	*SD:* Increased
Sengupta et al., 2020 [37]	*SD:* 110 (February to April 2020); April 2020: 50	*SD*: 33 (April 2019)	Burns-10 (9.1%) Hanging- 80 (72.7%) Poisoning-15 (13.7%) Others- 5 (4.5%)	NR	*SD*: Increased *The number of suicide cases during the first month of the lockdown following the pandemic has drastically increased compared to a couple of months prior
Shrestha et al*.,* 2021 [39]	*SD & Self-harm*: 55	2019; 38	Poisoning 47 (85.5%), Drug overdose 4 (7.3%), Hanging 4 (7.3%)	NR	*SD and Self-harm*: Increased
Yeates et al*.,* 2021 [38]	*SA:* 125 (1.9%)	2019; 120 (1.6%)	NR	NR	*SA:* No change

*SD* Suicidal deaths, *SA* Suicidal attempts, *NR* Not reported

## Risk factors associated with suicide/suicidal attempts during COVID-19 pandemic

Out of 18 studies included in this systematic review, only six studies [22, 23, 27, 30, 33, 39] provided data on associated factors for suicide and/or suicidal attempts during the COVID-19 pandemic. The mental health impact of social distancing was reported as an associated factor by Ammerman et al., 2021, while COVID-19 quarantine was mentioned as an associated factor identified by Daly et al., 2021. Three studies [23, 27, 39] reported that financial crises due to loss of employment are associated with suicide and/or suicidal attempts during COVID-19. Table 3 shows the reported associated factors of suicide/suicide attempts during the COVID-19 pandemic.

**Table 3:** Risk factors associated with suicidal deaths and suicidal attempts

Study	Reported risk factors
Ammerman et al., 2021 [30]	General distress Physical safety concerns Mental health impact of social distancing
Bergmans and Larson 2021 [22]	Male sex Aged group of 18–65 years Having a history of three or more encounters of suicide attempt or intentional self-harm Unmarried
Choudhury, 2020 [23]	Financial losses/job loss Domestic conflicts & violence Poverty and hunger Anxiety and depression
Daly et al., 2021 [33]	COVID-19 quarantine Pre-existing mental health condition/s
Nomura et al., 2021 [27]	Gender-based violence Loss of employment
Shrestha et al., 2021 [39]	Disputes with family members Economic crisis

## Recommendations for prevention of suicide/suicidal attempts during the COVID-19 pandemic

Recommendations for preventing suicide/suicide attempts were provided in nine studies [23, 25, 27, 28, 30, 31, 33, 35, 37]. Of them, we have identified common thematic recommendations for preventing suicidal deaths and suicidal attempts, and those were; 1) Develop a systematic suicide screening process and increase the suicide risk screening [23, 25, 28, 30, 37]. 2) Facilitate communication and increase access to the interventions for the people at risk [30, 35], 3) Design, develop, and provide interventions for mental health and psychological well-being for the people at risk (including mental health awareness programs, promoting social connectedness) [23, 30, 31, 33, 35, 37] 4) Implement measures to mitigate the impact on the economy (e.g., Provision of economic supports and changes in payment policies) [35, 37], and 5) Regulation of media reporting [27, 37]. The study done by Nomura et al., 2021 in Japan revealed an increased rate of suicide among women. Therefore, they recommended feasible ways of strengthening the financial condition of women by providing direct income support, cut down of tax, postponement or exemption of social security payments for temporary workers, support for women's income security to end the gender pay gaps and regulations to correct the under-valuation of women’s work, and provision of paid leave and flexible working arrangements [27]. Ammerman et al., 2021 stress the need of empowering suicidal risk screening to identify those who are at risk [30]. One potential option to facilitate large-scale risk detection is to incorporate suicide risk screenings into the protocol at COVID-19 testing sites, as the routine screenings are not possible due to the social distancing and stay-at-home regulations. This may be accomplished by including one- or two-question screener that would result in a follow-up phone call to facilitate care linkage if a suicide risk is recognized [30]. Recommendations for preventing suicide/suicidal attempts during the COVID-19 pandemic suggested by individual studies are shown in Table 4.

**Table 4:** Recommendations for preventing suicidal deaths and suicidal attempts

Study	Recommendations
Ammerman et al., 2021 [30]	− Increase suicide risk screening to identify those who are at risk − Introduce a more systematic screening process (e.g. integrate suicide risk screenings into the protocol at COVID-19 testing sites) − Implementation of follow-up phone calls to facilitate care connection, if suicide risk is indicated − Increase access to intervention for those who are experiencing psychological distress related to COVID-19
Carlin et al., 2021 [31]	− Consideration of mental health and psychological well-being when establishing lockdown policies
Choudhury, 2020 [23]	− Develop strategies and implement appropriate and timely interventions to eliminate the contributing predisposing factors to suicide − Implementation of community-based gatekeeper training programmes for early identification of suicidal ideations − Improvement of mental health awareness of individuals by the government along with the help of various NGOs − Strengthening of suicide screening services in the health care sector
Daly et al*.,* 2021 [33]	− Implementation of evidenced based strategies to minimize the risk of mental health deterioration associated with COVID-19 quarantine
Holland et al*.,* 2021 [35]	− Provision of counselling for those who are presented to emergency departments with risk of suicide/suicide attempts − Make linkage with existing behavioural health and social support services to provide immediate support for those who are in crisis − Conversion of existing in-person services health and social service to virtual means − Implementation of mass media campaigns that emphasize resilience, help-seeking, and available resources − Provision of economic supports to minimize financial stress, changes in payment policies − Regulatory changes to support telehealth − Promoting social connectedness
Nomura et al., 2021 [27]	− Implementation of immediate measures to mitigate the negative economic impact of COVID-19 on women − Strengthening virtual linkages for social support and mental health care delivery utilizing virtual platforms like video calls, telephones and social media − Regulation for media reporting to ensure the avoidance of fear and hopelessness among people
Radeloff et al*.*, 2021 [28]	− Careful monitoring of the suicidal rate as the COVID-19 crisis progresses in order to establish an evidence base prevention approaches
Sengupta et al., 2020 [37]	− Increase public awareness on how to deal with pressure and anxiety during the COVID-19 crisis − Implementation of targeted mental health surveillance of population at risk (e.g. patients with prior mental health diagnosis and older adults) − Provision of financial grants for food and unemployment support by the government − Refrain from irresponsible media reporting of suicide and highlight the precise facts about the causes and circumstances of suicide with due consideration to mental health problems − Establishment of policies/regulations for media reporting regarding reporting of such suicidal deaths
Isumi et al., 2021 [25]	− Close monitoring of suicide rates in children as the COVID-19 crisis in order to implement preventive measures, particularly after the reopening of schools

## The quality of the included studies

**Fig. 2:**
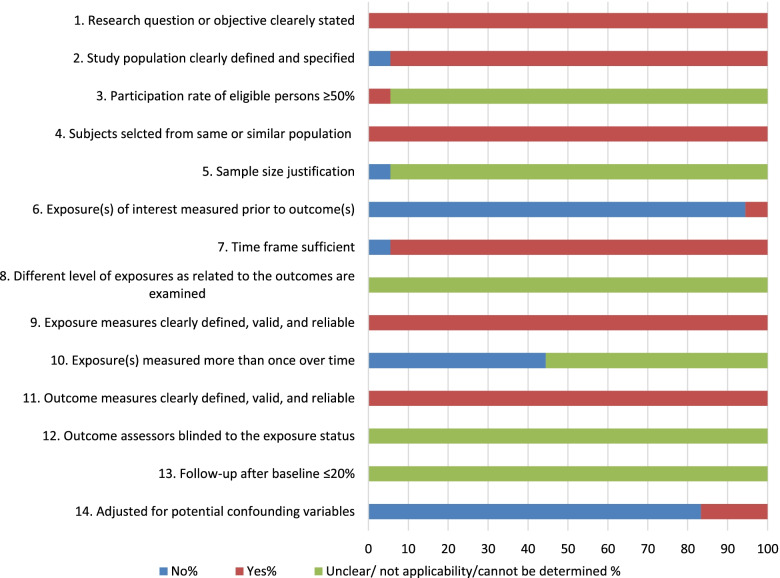
Quality assessment of included studies

In terms of the quality of included studies, six criteria out of 14 (43%) were satisfied by almost all of the included studies. Those criteria were; clearly defined research objective/s, clearly defined study populations, selection of subjects from a similar population, sufficient time frame, clearly defined exposure measures and clearly defined outcome measures. More than 80% of the studies have not measured the exposure before outcomes and adjusted for confounding variables. More than 40% of studies measured the exposure more than once over a time. Lastly, there were five criteria (36%) that we could not determine the quality or not applicable to assess the particular criteria in almost all the studies. Those criteria were; over ≥ 50% participation rate of the eligible person, having a justified sample size, having a different level of exposures as related to the outcomes examined, having blinded assessors of outcome/ s to the exposure status, and having a ≤ 20 follow-up from baseline (Fig. 2).

## Discussion

This systematic review presents several vital factors based on more than 12,746 suicidal attempts and 33,345 suicidal deaths reported during the COVID-19 pandemic. Suicidal death is a preventable loss that disturbs families, communities, and countries. Globally, it is a significant public health problem, and more than 700 000 people die due to suicide every year [40]. The present review found that the majority of the studies reported an increasing trend of suicidal attempts during the COVID 19 pandemic compared to the rates reported before the pandemic. Nevertheless, a review done in 2020 to find the suicidal behaviors and ideation during emerging viral disease outbreaks before the COVID-19 pandemic found weak evidence to suggest a significant increase in suicidal attempts and deaths by suicide during emerging viral disease outbreaks [41]. Compared to the global past pandemics, COVID 19 has tricky and complex mechanisms that have facilitated its rapid and catastrophic spread worldwide [42]. It is considered the most severe pandemic of the twenty-first century [43]. The rise in community distress due to the unexpected spread and rise of covid-19 virus has led to a pandemic status, eventually may have caused for the increasing trend of suicidal attempts reported during the COVID 19 pandemic compared to similar pandemics the world experienced before.

According to the WHO, a prior suicide attempt in normal circumstances is considered the most critical risk factor for suicide in the general population. However, the present systematic review reported domestic conflicts and violence, financial loss/job loss, anxiety and depression, and pre-existing mental health condition/s were there among the identified risk factors for suicidal attempts and suicidal deaths during the COVID-19 pandemic.

The social restriction practices and policies imposed by different countries secondary to the COVID 19 pandemic might have negatively influenced the fore-said risk factors that has been indirectly led increased rates of suicidal attempts and deaths. Moreover, in the wake of COVID 19, millions of people lost their access to employment and experienced financial hardships in day-to-day life [44]. It was evident that financial/employment-related issues substantially contribute to 13% of suicide deaths [45]. On the other hand, job loss or financial hardships independently lead to significant and persistent increases in domestic violence [46]. Similarly, social isolation and stay-at-home rules imposed by many counties badly resulted in domestic violence, making survivors of domestic violence at risk for further violence and isolating them from networks of support [47]. Furthermore, it is evident that during pandemics, a considerable number of people present with anxiety and depressive symptoms though they do not have any pre-existing mental health conditions [8]. As a result of this, some experience post-traumatic stress disorders in due course of their lives, which can end up with suicidal attempts or deaths. Nonetheless, a delayed increase in suicide rates is possible following major disasters [48–50]. Therefore, preventing suicidal attempts and deaths in the context of COVID 19 is a critical public health priority.

Considering the importance of preventing suicidal attempts and deaths secondary to the COVID 19 pandemic, early detection and timely intervention for individuals with suicidal behaviors is crucial [51]. Unlike previous pandemics, COVID-19 is occurring in the modern digital world, where video conferencing and virtual healthcare provision are widely available [52]. Although some people experience suicidal ideas during this pandemic, they might not attempt to seek help because of fear that meeting a health care professional face-to-face might put them at risk of contacting COVID-19. In this regard, as several studies have already identified the positive effects of new technologies in combating and preventing suicidal behavior, virtual platforms can be used as an effective way of screening to identify the people those at risk at the early stages [51, 53]. To strengthen this approach, community-based training programs can be used periodically to approach the neediest people early. Moreover, media should also be responsible when reporting facts, avoiding stoking fear and hopelessness among people in the community [18]. Therefore, it is recommended to use the recommendations given by this review to prevent suicidal attempts or suicidal deaths during the COVID-19 pandemic.

There are a few limitations in this review. The findings of this review can be limited because of not including the potential articles beyond the search strategies. Besides, the studies included in this review used different durations for data collection during the COVID 19 pandemic making it difficult to estimate the incidence rate of suicidal attempts and deaths only for the COVID 19 pandemic. Moreover, the inter-rater agreement was not calculated for this review as the screening process was undertaken in Covidence systematic review manager software. Despite these limitations, this review provides; (i) the first observation of suicidal attempts and suicidal deaths during the COVID-19 pandemic, (ii) the risk factors for suicidal attempts and suicidal deaths during the COVID-19 pandemic, and (iii) the recommendations in preventing suicidal attempts or suicidal deaths during the COVID-19 pandemic. Suicide prevention in the COVID-19 era and similar pandemics are crucial and challenging. Therefore, it is essential to select appropriate suicide prevention strategies based on strong evidence. The findings of this study can be used when selecting appropriate suicide prevention strategies considering the identified risk factors and recommendations given. It is recommended to discuss the longitudinal trends in future studies.

## Data Availability

The data that support the findings of this study are available on request from the corresponding author.
